# Artificial Intelligence in Hospice Dementia care: Perspectives of Hospice Staff and Family Caregivers

**DOI:** 10.1093/geroni/igaf122.4068

**Published:** 2025-12-31

**Authors:** Oonjee Oh, George Demiris

**Affiliations:** University of Pennsylvania, Philadelphia, Pennsylvania, United States; University of Pennsylvania, Philadelphia, Pennsylvania, United States

## Abstract

Artificial intelligence (AI) has gained attention as a tool to support dementia care through efficient and personalized interventions. However, individuals situated at the intersection of dementia and end-of-life care are particularly vulnerable due to severe conditions and lack of decision-making capacities. Yet, there is limited research on AI acceptability in this specific context. Hence, we aimed to examine the perceptions of family caregivers and hospice staff regarding AI integration in end-of-life care settings for persons with dementia. We adopted a qualitative descriptive design with 19 participants (10 dementia caregivers in hospice and 9 hospice staff members). Dementia caregivers were interviewed individually and staff, including nurses, social workers, bereavement counselors, and a music therapist, participated in 3 focus group interviews. Transcripts were analyzed using a directed content analysis approach; the Technology Acceptance Model served as the guiding framework. We explored themes of “perceived usefulness,” “perceived ease of use,” and the inductive theme of “guiding the new wave of the future.” Caregivers and staff identified strengths and concerns of using AI in hospice dementia care. Example strengths included providing comprehensible informational support to the families and assisting with monitoring symptoms at home. Concerns included the perceived absence of human touch and warmth in AI, which may create resistance among families to adopt AI, and its potential lack of nuanced understanding of dementia-related behaviors. Staff also highlighted critical considerations when designing and deploying AI for this population, such as recognizing the heterogeneity in dementia and integrating the voices of those providing direct care.

